# Renal Tumors of Childhood—A Histopathologic Pattern-Based Diagnostic Approach

**DOI:** 10.3390/cancers12030729

**Published:** 2020-03-19

**Authors:** Ariadne H.A.G. Ooms, Gordan M. Vujanić, Ellen D’Hooghe, Paola Collini, Aurore L’Herminé-Coulomb, Christian Vokuhl, Norbert Graf, Marry M. van den Heuvel-Eibrink, Ronald R. de Krijger

**Affiliations:** 1 Princess Máxima Center for pediatric oncology, 3584 CS Utrecht, The Netherlandsm.m.vandenheuvel-eibrink@prinsesmaximacentrum.nl (M.M.v.d.H.-E.); 2 Pathan B.V., 3045 PM Rotterdam, The Netherlands; 3 Department of Pathology, Sidra Medicine, Doha 0000, Qatar; gvujanic@sidra.org; 4 Department of Pathology, Oslo University Hospital, Rikshospitalet, 0372 Oslo, Norway; eldhoo@ous-hf.no; 5 Department of Diagnostic Pathology and Laboratory Medicine, Fondazione IRCCS Istituto Nazionale dei Tumori, 20133 Milan, Italy; Paola.Collini@istitutotumori.mi.it; 6 Sorbonne Université, Department of Pathology, Hôpital Armand Trousseau, Hopitaux Universitaires Est Parisien, 75012 Paris, France; aurore.coulomb@aphp.fr; 7 Section of Pediatric Pathology, Department of Pathology, University Hospital Bonn, 53127 Bonn, Germany; Christian.Vokuhl@ukbonn.de; 8 Department of Pediatric Oncology & Hematology, Saarland University, D-66421 Homburg, Germany; Norbert.Graf@uks.eu; 9 Department of Pathology, University Medical Center Utrecht, 3584 CX Utrecht, The Netherlands

**Keywords:** pediatric, oncology, renal tumors, immunohistochemistry, molecular analysis

## Abstract

Renal tumors comprise approximately 7% of all malignant pediatric tumors. This is a highly heterogeneous group of tumors, each with its own therapeutic management, outcome, and association with germline predispositions. Histopathology is the key in establishing the correct diagnosis, and therefore pathologists with expertise in pediatric oncology are needed for dealing with these rare tumors. While each tumor shows different histologic features, they do have considerable overlap in cell type and histologic pattern, making the diagnosis difficult to establish, if based on routine histology alone. To this end, ancillary techniques, such as immunohistochemistry and molecular analysis, can be of great importance for the correct diagnosis, resulting in appropriate treatment. To use ancillary techniques cost-effectively, we propose a pattern-based approach and provide recommendations to aid in deciding which panel of antibodies, supplemented by molecular characterization of a subset of genes, are required.

## 1. Introduction

In Europe, each year about 1000 children are diagnosed with a renal tumor, thereby comprising approximately 7% of all pediatric malignant tumors. Approximately 90% of pediatric renal tumors comprise Wilms tumor (WT), the remainder consisting of clear cell sarcoma of the kidney (CCSK), malignant rhabdoid tumor of the kidney (MRTK), renal cell carcinoma (RCC), congenital mesoblastic nephroma (CMN), and other rare tumors (Table 1) [1]. They represent a very heterogeneous group, which includes a spectrum of tumors from those with low malignant potential (e.g., CMN) to highly aggressive tumors (e.g., MRTK) [2,3], requiring different treatment, and showing remarkable intra-tumor histologic and genetic heterogeneity [4,5]. In addition, it has been recognized that an increasing number of pediatric renal tumors have been associated with specific syndromes or diseases (Table S1) [6], which highlights the importance of precise tumor diagnosis, not only for appropriate treatment, but also for genetic counseling.

While imaging techniques are continuously improving, they are still unable to reliably diagnose and distinguish the most relevant pathology based risk groups, although some new techniques (like MRI-Diffusion Weighted Imaging) might be helpful in the near future [7]. The clinical characteristics of children with renal tumors are similar, although some (like age distribution) may be helpful in the differential diagnosis [8].

In nearly all tumors, the final diagnosis and classification of clinically relevant (sub)types still relies on histopathologic examination. Updated pathology guidelines for handling renal tumors have been published recently by the Renal Tumor Study Group of the International Society of Paediatric Oncology/Société Internationale D’Oncologie Pédiatrique (SIOP-RTSG) in the SIOP-RTSG UMBRELLA 2016 protocol [9]. These guidelines recommend that tumors should be extensively sampled, to allow diagnostic classification, staging, and to capture tumor heterogeneity [9]. Difficulties in achieving the correct histologic classification may arise due to the ability of tumors to display a variety of overlapping histologic patterns. This stresses the need for adequate tumor sampling, since it can reveal other important histopathologic clues. Biopsies, performed in selected cases only, might pose even more difficulties, due to the limited availability of material [10]. Finally, in some cases where abdominal tumors are so large that the kidney and adjacent organs are unrecognizable, even non-renal tumors have to be included into the differential diagnosis. In many cases, additional ancillary techniques are needed to establish the final diagnosis. Since clinically relevant discrepancies between the diagnosis and/or staging of institutional pathologists and central pathology review persist [11,12], rapid central pathology review is now mandatory in both the Children’s Oncology Group (COG) and SIOP studies, in order to establish the correct diagnosis and stage within a clinically relevant timeframe and start appropriate treatment.

In this review, to aid in the diagnostic process, we use a pattern-based diagnostic approach for the most common pediatric renal tumors, by discussing pattern-based differential diagnoses and in providing recommendations for the use of ancillary techniques.

## 2. A Pattern-Based Approach to the Use of Ancillary Techniques in Pediatric Renal Tumors

**Figure 1:**
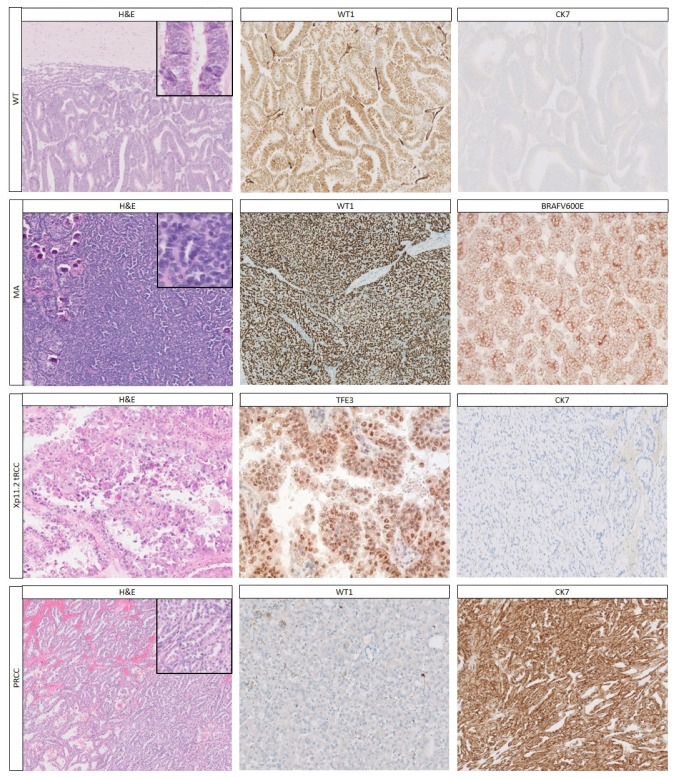
The most common pediatric renal tumors with an epithelial pattern and their most important immunohistochemical staining. Epithelial Wilms tumor (WT) showing epithelial structures which shows characteristic strong nuclear staining for WT1 as well as lack of staining for CK7. The classical histology of metanephric adenoma (MA) consists of closely packed mature tubules and papillary structures as well as psammoma calcifications, which shows strong nuclear staining for WT1, but also strong staining for BRAFV600E corresponding to the *BRAF* mutation present in ~80–90% of MAs. Xp11.2 translocation renal cell carcinomas (tRCCs) typically show large epithelioid cells with copious amounts of eosinophilic cytoplasm and a large eosinophilic nucleolus; these tumors show nuclear TFE3 staining and most cases show absent or decreased staining for cytokeratins, such as the negative cytokeratin 7 (CK7) shown here. Papillary renal cell carcinoma (PRCC) with typical papillary structures, often with rather small monotonous nuclei, with lack of reactivity to WT1, but strong staining for CK7. H&E and immunohistochemical stainings at 10× magnification; inset at 40× magnification.

**Figure 2:**
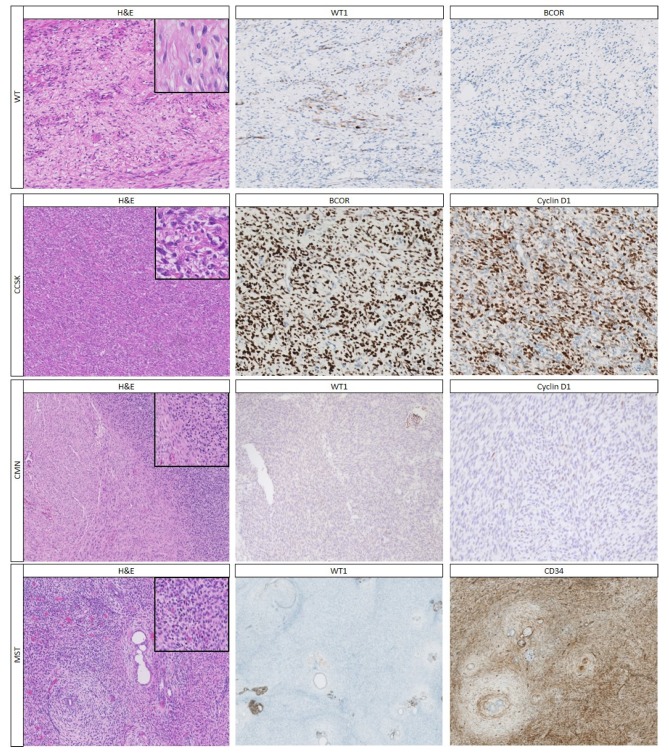
The most common pediatric renal tumors with a stromal pattern and their most important immunohistochemical stainings. The stromal type WT consists of spindle cells, which often shows more patchy or focal staining for WT-1 and they lack staining for BCL6 corepressor (BCOR). The classical pattern of a clear cell sarcoma of the kidney (CCSK) has a fine vasculature surrounding nests of rather monotonous cells (“chicken wire” vasculature), and the characteristic strong nuclear staining for BCOR as well as CyclinD1. A mixed type congenital mesoblastic nephroma (CMN) with hypercellular areas resembling the cellular subtype and less cellular areas resembling the classic subtype; the immunohistochemical pattern is rather indistinct, including lack of staining for WT1 and CyclinD1. Metanephric stromal tumor (MST), showing the formation of concentric rings of stroma surrounding some of the epithelial tubules (referred to as “onion skinning”); the stromal component of metanephric tumors lacks WT1 staining, but stains strongly with CD34. H&E and immunohistochemical stainings at 10× magnification; inset at 40× magnification.

**Figure 3:**
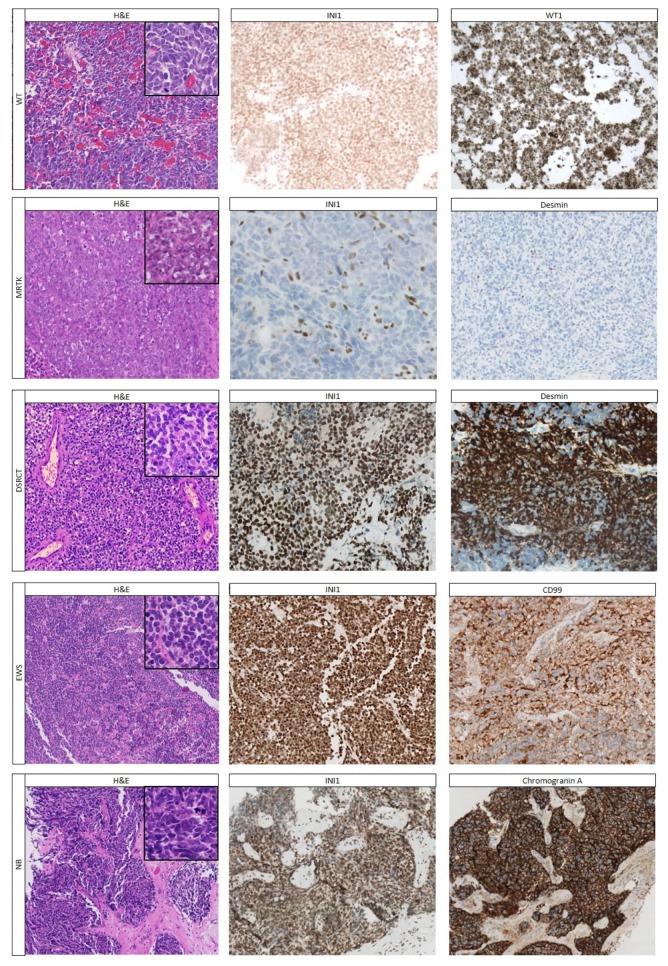
The most common pediatric tumors with an undifferentiated pattern and their most important immunohistochemical stainings. Wilms tumor (WT) with undifferentiated blastema shows strong nuclear staining for INI1 as well as for WT1. Malignant rhabdoid tumors of the kidney (MRTKs) often show large cells with large eccentric nuclei, characteristic loss of INI1 staining and a significant percentage of MRTKs do not stain (or only focally) for muscle markers such as desmin. The rare desmoplastic small round cell tumor (DSRCT), depicted in the third row, highly resembles blastemal WTs specifically with staining for WT1 (only C-terminal clone; not shown) and MRTKs, but with strong staining for INI1 and desmin in all cells. Ewing sarcoma shows INI1 staining and strong membranous CD99. When tumors are large and infiltrate the kidney, tumors such as neuroblastoma (NB) enter the differential diagnosis. As depicted in the last row, these also show a small blue round cell phenotype, without loss of INI1, but with strong staining for neuroendocrine markers such as chromogranin A. H&E and immunohistochemical stainings at 10× magnification; inset at 40× magnification.

In general, there are three major histologic patterns which are the basis for the differential diagnoses of any tumor, including pediatric renal tumors: an epithelial, a mesenchymal, and an undifferentiated pattern (Table 2, Table 3 and Table 4). Most tumors harbor histological clues to the diagnosis, although these can be present only focally. In WTs, all three patterns are often present, but many WTs show only one or two patterns (Figure 1, Figure 2 and Figure 3), or show the other patterns only focally. This underlines the reasons why extensive sampling is required, in order to capture all tumor features [9]. The most common pediatric renal tumors have been described and reviewed extensively elsewhere [3,13,14,15,16], so we only briefly recapitulated their main histologic features (Table S2). Since some tumors harbor a variety of histologic patterns, they appear in more than one of these categories.

### 2.1. Epithelial Pattern

#### 2.1.1. Histology

Tumors demonstrating a pure epithelial pattern include epithelial type WT, hyperplastic perilobar nephrogenic rests, RCC and metanephric adenoma (MA), with WT being by far the most common one.

Epithelial WTs (Figure 1) usually occur in younger children (median age 15 months), and about 80% of cases are stage I [54]. The tumor consists of epithelial structures, which may demonstrate the whole spectrum of nephrogenesis, varying from poorly and moderately differentiated to mature tubules and glomeruli-like structures. Tumor cells are often columnar with elongated, hyperchromatic and crowded nuclei, inconspicuous nucleoli, and scant cytoplasm. Mitoses are often easily identified. For WTs that consist entirely of poorly and moderately differentiated tubules, the diagnosis is more difficult, and tumors, such as papillary RCC and MA, have to be excluded.

If nephrogenic rests (NRs) are identified, the diagnosis of WT can be reliably established since NRs are virtually never associated with non-WTs [55]. NRs are foci of embryonal cells that persist after 36 weeks of gestation and they are considered precursors of WTs [56]. NRs accompany WTs in approximately 40% in unilateral WTs and in >90% of bilateral WTs [56,57]. NRs are subdivided into two main subtypes: perilobar nephrogenic rests (PLNRs) and intralobar nephrogenic rests (ILNRs). PLNRs are confined to the periphery of the renal lobe, while ILNRs can be found anywhere within the renal lobe [56,58]. Nephroblastomatosis is defined as the “diffuse or multifocal presence of NRs” [56]. Hyperplastic NRs can be difficult to distinguish from WTs, as there are no histologic, immunohistochemical or molecular features that are helpful, so the diagnosis is commonly based on experts’ consensus. The presence of a fibrous capsule between the lesion and renal parenchyma may be helpful in diagnosing a WT in patients treated with primary surgery, but not in those treated with preoperative chemotherapy, where a pseudocapsule is often found around NRs (GMV, personal observation).

RCC is the most important tumor in the differential diagnosis of epithelial WT, particularly in older children [27]. Overall, RCCs account for only ~2–4% of pediatric renal tumors, but this increases to >50% in children older than 12 years [27]. The most prevalent RCC types in adulthood (e.g., clear cell RCC, papillary RCC) are less common in childhood [27], but it is papillary RCC (PRCC) which needs to be considered in children in the differential diagnosis of epithelial WTs, due to the overlapping histologic architecture and cell type. PRCCs can be separated into Type 1 and Type 2. Type 1 is composed of small cuboidal cells with scant pale cytoplasm arranged as single layers on papillae, tubules and glomerulus-like structures, and often contain foamy macrophages and psammoma bodies, in contrast to WTs. Type 2 PRCCs show papillae lined with pseudostratified cells, abundant eosinophilic to occasionally clear cytoplasm, and atypical nuclei with prominent nucleoli, which is not a common feature in WT. PRCCs usually have no pseudocapsule, and lack a blastemal and stromal component.

In children, the Microphtalmia-associated Transcription factor family translocation RCC (MiT-RCC) is the most common RCC subtype (42% of all pediatric RCCs), comprised of Xp11.2 tRCC (~90% of cases) and t(6;11) RCC [27]. Since these tumors have distinct histologic characteristics (Table S2), they are usually not difficult to diagnose and distinguish from WT, but the characteristic features can be present only focally, and may thus, be easily overlooked [14].

In addition, there are some RCC subtypes, which are generally rare, with very few cases reported in children. Still, they are important to recognize, since some of them are associated with germline mutations or syndromes, including Anaplastic Lymphoma Kinase (ALK)-rearranged-RCC (ALK-RCC), Succinate Dehydrogenase-deficient renal cell carcinomas (SDH-RCC), and Hereditary Leiomyomatosis and Renal Cell Carcinoma-RCC (HLRCC-RCC) [59]. Most of these tumors show characteristic histologic features (Table S2), although these can be present only focally, and immunohistochemistry and molecular analysis will aid in establishing the correct diagnosis.

Metanephric adenoma is another tumor which can be difficult to distinguish from WTs and RCCs, especially in older children. Histologically, MAs consist of small, uniform closely packed tubules and papilla (Figure 1). Features which can aid in distinguishing them from WTs are the absence of a pseudocapsule, sharp demarcation from the renal parenchyma, the presence of small uniform cells with scant cytoplasm, bland nuclei, fine chromatin, absence of atypia, psammoma bodies, and rare mitoses (in epithelial WTs mitoses are more common) [17,60]. Nevertheless, in a recent report, tumors with histologic and molecular overlap between MA and epithelial WTs were described [61].

Other types of RCC that are more common in adulthood and rare in children are extensively described in literature and not further described here [62,63,64,65]. Finally, in addition to tumors that are genuinely epithelial, there are tumors that can have an epithelioid appearance, such as MRTKs, CCSKs (see below), and epithelioid angiomyolipoma. However, these tumors have other features (such as fat and/or aberrant vessels in epithelioid angiomyolipomas), which distinguish them from tumors with a genuine epithelial pattern.

#### 2.1.2. Immunohistochemistry

Immunohistochemistry using a small panel of immunohistochemical markers (Table 2) including WT1, pan-cytokeratin (pan-CK), and CK7 is helpful in reaching a definite diagnosis in most cases. Nevertheless, immunohistochemistry does not reliably differentiate between NRs and WTs.

In pure epithelial WTs, WT1 is positive in 80–90% of WTs, while all RCC types are negative for WT1 [19,40]. Translocation RCCs are often only weakly positive for keratins, but strongly positive for melanocytic markers (TFEB-tRCCs more commonly and more diffusely than TFE3-tRCCs). Xp11.2 RCC and t(6;11) RCCs show nuclear TFE3 (Figure 1), and TFEB positivity, respectively [14,66]. Notably, a diagnostic pitfall in TFE3 positive RCCs, is that the rare ALK-RCCs also stain positive [67]. Depending on the RCC subtype, further immunohistochemical stains (e.g., CK7, AMACR, CD10, vimentin, cathepsin K) will help establish the diagnosis and rule out WT [20,22,29,68].

If both WT1 and pan-CK are positive, the main differential diagnosis remains between epithelial WT and MA. The majority (~90%) of MAs carry a *BRAF* mutation (Table 5), and the most common one (*BRAFV600E*) can be demonstrated by immunohistochemistry (see also below). Interestingly, a recent study showed *BRAF*V600E staining in both MA and epithelial WTs with overlapping features [61]. Additionally, diffuse CD57 staining favors the diagnosis of MA, but does not rule out WT [20], whereas CK7 negativity of both WTs and MAs distinguishes them from PRCC [17,20].

In general, all other tumors which can show an epithelioid pattern (MRTKs, CCSKs, epithelioid angiomyolipomas) are negative for WT1 [40]. To further distinguish between these tumors, the panel of antibodies can be extended (Table 6), for example with PAX8 for the distinction of TFE-tRCCs from epithelioid angiomyolipomas, since the latter are also positive for melanocytic markers and angiomyolipomas may also have Xp11 (*TFE3*-related) translocations (next sections; Table 7).

#### 2.1.3. Genetics

Genetic analysis plays a minor role in the identification of WTs, since >50 driver mutations are known in WTs (Table 5), but the most commonly mutated genes may be analyzed to exclude other entities [69]. Interestingly, *TRIM28* mutations have been associated with a specific subset of purely epithelial WTs [70].

Type 1 PRCCs show *MET* alterations in 81%, and 25% of type 2 PRCCs harbor *CDKN2A* genomic abnormalities [71]. MiT-RCCs harbor translocations that lead to fusions with different genes: Xp11.2 tRCCs harbor translocations of the Transcription Factor E3 (*TFE3*) gene with different fusion partners (Table 7), and t(6;11) RCC fuses Transcription Factor EB (*TFEB*) to *MALAT1*. Since some antibodies can be technically challenging and sensitive to suboptimal fixation (e.g., TFE3 and TFEB) [72], Fluorescent in situ hybridization (FISH) break apart probes detecting the *TFE3* [73], *TFEB* [74], and *ALK* translocation [67,75,76] can be used (Table 7), although false negative results have been reported.

### 2.2. Mesenchymal Pattern

#### 2.2.1. Histology

Mesenchymal tumors generally consist of spindle cells arranged in different patterns, such as short or long bundles, fascicles or storiform architecture. WTs can consist (nearly) completely of mesenchymal cells (Figure 2), and thorough sampling can help revealing important histologic clues to the diagnosis of WTs (such as the finding of NRs or small foci of other WT components). CCSK, CMN and metanephric stromal tumor (MST) are the most important differential diagnostic considerations. In rare cases, sarcomas (e.g., synovial sarcoma), inflammatory myofibroblastic tumors, and RCCs with sarcomatous differentiation enter the differential diagnosis.

Histologically, different patterns exist in CCSKs, leading to a substantial number of misdiagnoses [38]. Nevertheless, approximately 90% of CCSKs show, at least focally, the classic pattern with bland ovoid cells with monomorphous nuclei with finely dispersed chromatin and small nucleoli, which are arranged in cords and nests separated by a chicken-wire vasculature (Figure 2) [13,38].

In very young children, CMN is another important consideration. It encompasses three histologic variants: classic, cellular, and mixed, containing areas of both cellular and classic histology (Table S2). The classic type consists of bland spindle cells organized in an interlacing fascicular pattern, embedded in collagenous stroma blending into the pre-existent renal parenchyma, with entrapment of normal renal parenchyma. The cellular type has a higher cellularity of ovoid cells with less cytoplasm, organized in solid sheets, and with a higher proliferation rate, and a rather clear border towards the renal parenchyma [3].

MST is a rare tumor, which histologically resembles CMN, but occurs at an older age than CMN. It shows some characteristic histologic features, including alternating cellularity, a scalloped border with the renal parenchyma, perivascular cuffing, and angiodysplasia (Table S2; Figure 2) [42].

When the most common renal tumors showing a mesenchymal pattern are excluded, some very rare tumors, such as primary renal sarcomas need to be considered, especially in older children [77]. Interestingly, primary renal synovial sarcomas are usually monophasic [77]. Although, rare in the kidney, inflammatory myofibroblastic tumor is another diagnostic consideration.

Finally, in teenagers and young adults, sarcomatous dedifferentiation of RCC types (such as clear cell RCC) is a phenomenon to consider in the differential diagnosis. Interestingly, in these spindle cell areas, immunoreactivity for markers normally seen in the original tumor type is often lost, whereas most cases show abnormal p53 immunoreactivity suggestive of a *TP53* mutation [78]. Additional sampling in search for the original tumor type is warranted.

#### 2.2.2. Immunohistochemistry

Although, many renal tumors with a mesenchymal pattern lack a characteristic immunohistochemical profile (Table 3), a panel of markers can be of added value to differentiate the tumors (Table 3, Table 6). In the stromal component of WTs, nuclear staining for WT1 is often weak or absent. CCSKs show a distinctive immunohistochemical profile, with strong CyclinD1, BCL6 corepressor (BCOR), and NGFR staining, and negative WT1 staining (Figure 2; Table 3) [31,32,33,34,39]. CMNs have a rather non-specific immunohistochemical pattern (Figure 2; Table 3), although pan-Trk staining can be used to diagnose cellular CMNs harboring the *ETV6-NTRK3* translocation (see below) [79]. CMNs may show variable CyclinD1 positivity [31,32], and in very rare cases even WT1 positivity [40]. The majority of MSTs are BRAFV600E (~65%) and CD34 positive (Figure 2), while the other tumors are usually negative [18,35,42,80]. The rare primary renal synovial sarcomas are positive for CD99, vimentin and TLE1, and often demonstrate at least focal expression of cytokeratins and EMA, but can be negative or only weakly positive for INI1 [81]. Furthermore, nearly all stromal WTs and synovial sarcomas show strong BCL-2 staining, whereas approximately half of CCSK and all CMNs are negative [35].

#### 2.2.3. Genetics

If histologic and immunohistochemical features are insufficient to reach a final diagnosis, molecular analysis can help in the distinction of some mesenchymal tumors (Table 5 and Table 7). As mentioned in previous sections, many driver genes are involved in WTs (Table 5), which may be included in a targeted sequencing panel. In CCSKs, ~85% harbor the *BCOR*-internal tandem duplication (*BCOR*-ITD) of exon 15, and ~10% show the t(10;17) leading to *YWHAE-NUTM2* fusion. These two molecular abnormalities are mutually exclusive and can be demonstrated by FISH and/or sequencing techniques [82,83]. Rare CCSKs harbor the *BCOR-CCNB3* fusion [39]. The recently reported *EGFR*-ITDs rearrangements in all CMN types, together with the previously known t(12;15) (*ETV6-NTRK3* translocation) in cellular CMNs help to confirm the diagnosis of CMN [84,85]. *BRAF V600e* mutations in MSTs can be identified by immunohistochemistry or sequencing [86]. A PCR study on 22 CMNs (including 10 cellular CMNs) showed no *BRAF* V600e mutations [80]. In one study 2 out of 3 cellular CMN which showed no *ETV6* translocation, showed different rearrangements in the *BRAF* oncogene on WGS [84]. The diagnosis of synovial sarcoma can be established by demonstrating the characteristic translocation t(X;18)(p11.2;q11.2), present in >90% of cases [87].

### 2.3. Undifferentiated Pattern

#### 2.3.1. Histology

Some pediatric renal tumors have an undifferentiated “small round blue cell” appearance, such as blastemal WTs, MRTKs, as well as tumors that rarely involve or infiltrate the kidney, such as (poorly differentiated/undifferentiated) neuroblastoma, desmoplastic small round cell tumor (DSRCT), and Ewing sarcoma (EWS) (Table S2). In addition, lymphoma should always be considered and excluded. Other tumors described above (e.g., CCSK) occasionally show an undifferentiated appearance.

The blastemal WT component consists of undifferentiated, small blue round cells with a high cellularity of round to ovoid cells with a high nuclear–cytoplasmic ratio, and brisk mitotic activity (Figure 3). Blastema often shows different histologic patterns, such as nodular, trabecular, diffuse, and serpentine [88].

MRTKs are aggressive infiltrating tumors with a very poor prognosis, often presenting in patients younger than two years of age [2,15]. They are highly cellular, non-encapsulated tumors composed of infiltrating sheets of non-cohesive cells with a large eccentric nucleus, and abundant eosinophilic cytoplasm (Figure 3) (Table S2) [15]. However, MRTKs may show different patterns, which sometimes lead to difficulties in achieving a correct diagnosis on routine H&E staining only [15].

Neuroblastomas often originate in the adrenal gland, but very rarely in the kidney. Clinically, most neuroblastomas can be suspected by elevated levels of catecholamines in urine and serum. Histologically, the nuclei of the undifferentiated cells show the characteristic coarse ‘salt and pepper’ chromatin. Neuroblastomas lack a pseudocapsule, and often show neuroblasts exhibiting variable degrees of differentiation up to ganglion cells and neuropil, which is the basis for histologic classification [89].

DSRCTs have been rarely described in the kidney [90]. Histologically, they consist of solid nests of round to oval cells and show necrosis and occasional pseudoglandular-/rosette formation [44,91]. Intriguingly, renal DSRCTs show no desmoplasia, making their histologic diagnosis even more difficult [90].

Primary renal sarcomas rarely occur, and are histologically characterized by undifferentiated morphology. EWS can originate in the kidney or develop in the retroperitoneum with infiltration of the kidney [45,92]. These tumors consist of small round to oval cells with scanty cytoplasm, molding, high mitotic activity, and with condensation around blood vessels [45]. Two newly described sarcomas, very rarely seen in children in the kidney, are the *CIC-DUX4* translocation sarcoma and the *BCOR-CCNB3* translocation sarcomas [93]. Both tumors often show a spectrum of round cells to more epithelioid and spindle cells, often present at least focally [93,94]. The *CIC-DUX4* sarcomas behave highly aggressively, often metastasizing early in the disease course [93,94].

Renal medullary carcinoma (RMC) and collecting duct carcinoma (CDC) may show significant histologic overlap with MRTKs. Helpful diagnostic clues are the presence of a reticular or cribriform pattern and finding of sickle cells in RMC [95], while dysplastic changes in the adjacent tubules and a dense inflammatory infiltrate suggest CDC [95,96].

#### 2.3.2. Immunohistochemistry

The differential diagnosis of “small blue round cell tumors” of the kidney can be difficult, but most of these tumors can be classified using ancillary techniques (Table 4 and Table 5). The blastemal component of WTs demonstrates nuclear WT1 positivity in 80% of cases [17,46]. DSRCTs are also WT1 positive [44], but only with the C-terminal clone of WT1, while WTs are positive, with both the N-terminal and C-terminal clone [40,44,47,48]. DSRCTs also show dotlike cytokeratin and desmin positivity [16,44]. Blastemal type WTs and neuroblastomas are both positive for CD56, but WT1 is negative in neuroblastoma [16,40,47], while synaptophysin, chromogranin A, NB84, and PHOX2B are positive in neuroblastoma [49,53]. Furthermore, WTs are PAX8 positive, while neuroblastoma, DSRCT and sarcoma are PAX8 negative [34]. MRTKs can be rather easily distinguished from all other pediatric renal tumors by their complete loss of INI1 staining (Figure 3) and PAX8 negativity.

However, RMCs can also show near complete loss of INI1 immunoreactivity, but are PAX8 positive [16,52,96,97]. In EWS, CD99 (diffuse strong membranous) and cyclinD1 (nuclear), in combination with lack of reactivity to WT1, discriminates it from WT and almost all other tumors [16,51]. Of note, *CIC-DUX4* translocation sarcomas have been reported to show WT1 staining in >90%, while *BCOR*-translocation sarcomas were reported to show strong staining of BCOR, BCL-2, cyclinD1, and TLE1 staining in most cases (each 80–90%) [93,94]. Further differentiation of undifferentiated round cell sarcomas needs molecular studies (below).

#### 2.3.3. Genetics

In difficult cases, next generation (targeted) sequencing can be performed including genes frequently mutated or altered in WTs (e.g., *WTX, WT1, CTNNB1, SIX1, SIX2*, miRNA processing genes) and neuroblastomas (*PHOXB2*, *ALK*, *ARID1A/B* genes) (Table 5). Notably, *MYCN* can show alterations (mutations as well as gains) in both WT and neuroblastoma [98].

MRTKs are characterized by inactivating mutations in the chromatin-remodeling complex members *INI1* (also referred to as *SMARCB1*; 95%) or *SMARCA4* (5%), or show deletions of 22q11 (on which *INI1* is located) [99,100,101]. Therefore, sequencing of *INI1* and *SMARCA4* is helpful in establishing a diagnosis of MRTK. Notably, when patients with a MRTK have a synchronous or metachronous rhabdoid tumor affecting other organs (including the central nervous system, referred to as Atypical Teratoid/Rhabdoid Tumor (AT/RT)), it is highly suggestive of the rhabdoid tumor predisposition syndrome [102].

The diagnosis of DSRCT is based on the demonstration of the characteristic t(11;22)(p13;q12) leading to *EWS-WT1* fusion [90]. In contrast, EWS show a wide variety of translocations involving the EWS gene (Table 5 and Table 7), most commonly t(11;22)(q24;q12) resulting in the *EWS-FLI1* fusion [45,92]. In *EWS*-negative round cell sarcomas, C*IC-DUX4* gene fusion (resulting from either t(4;19) or t(10;19)), is the most common genetic abnormality detected [94]. The remainder includes *BCOR-CCNB3* translocation sarcomas, harboring the same translocation reported in some CCSKs, suggesting that they might be in the same spectrum [93,103].

### 2.4. Biphasic Tumors

In the differential diagnosis of WTs with stromal and epithelial components, Metanephric adenofibroma (MAF) needs to be considered, despite its rarity. MAFs show features of both MST and MA (as described above), including positive nuclear staining for WT1 in the epithelial component, and positive CD34 staining in the stromal component [104,105]. Furthermore, approximately 50% of MAFs carry the *BRAF V600E* mutation, although rare WTs may also harbor a *BRAF* mutation [18,61,106].

## 3. Future Perspectives

In addition to Hematoxylin and Eosin (H&E)-based diagnosis, with the aid of immunohistochemistry and targeted molecular analysis, there are now several developments, which may revolutionize tumor diagnosis in the near future. First of all, genome-wide molecular methods, including whole exome sequencing, RNA sequencing, and methylation arrays, allow unprecedented molecular classification of tumors, as has been shown for brain tumors and renal cell carcinoma [107,108]. While tumor heterogeneity, which is undoubtedly present in Wilms tumors, might be an obstacle to these genome wide molecular methods, liquid biopsy holds the promise that it would be able to circumvent this problem, as cell-free DNA in the blood might be representative of the entire tumor [109]. Another strategy, by which pediatric renal tumors might be classified, is based on recent advancements in the field of radiology, especially the addition of diffusion-weighted imaging to standard magnetic resonance imaging, with which apparent diffusion coefficient metrics may predict the composition of renal tumors, especially that of WTs [110]. Finally, through digitalization of traditional H&E slides, so-called whole slide images can now be analyzed by machine learning techniques. This has already resulted in automated recognition of specific tissue components, such as lymph node metastases in breast cancer [7]. It is certainly conceivable that such automated recognition will also lead to computer-assisted classification of tumor types, including pediatric renal tumors.

## 4. Conclusions

The differential diagnosis of pediatric renal tumors can be challenging if based solely on H&E staining, due to the presence of similar cell types and overlapping histologic patterns across these tumors. Extensive sampling may reveal the presence of distinctive histologic features, allowing a diagnosis or narrowing the differential diagnosis. However, the use of ancillary techniques is necessary in many cases. Many ancillary techniques are integrated now in daily pathology practice and provide the pathologist with tools to achieve the correct diagnosis, which contributes to multidisciplinary decision-making regarding the appropriate treatment and counselling for each child with a renal tumor. Since there is a large repertoire of immunostainings and genetic aberrations, the challenge lies in choosing a restricted panel of ancillary techniques, which results in an appropriate diagnosis in an acceptable time frame and at an acceptable cost. In this review, we used a pattern-based approach to discuss the differential diagnosis, and provided recommendations for panels of ancillary techniques. The focused use of ancillary techniques will likely result in fewer unclassified cases, and lead to an increase in children diagnosed with germline mutations. This will result in patients benefitting from receiving adequate therapies and counseling.

## Figures and Tables

**Table 1:** Distribution of pediatric renal tumors in children <15 year of age according to the National Registry Childhood Tumors (UK).

Tumor Type	Percentage (%)
**WT**	88.0
**CCSK**	3.6
**MRTK**	3.0
**CMN**	3.0
**RCC**	1.8
**Others**	0.6

WT = Wilms tumor; CCSK = clear cell sarcoma of the kidney; MRTK = malignant rhabdoid tumor of the kidney; CMN = congenital mesoblastic nephroma; RCC = renal cell carcinoma.

**Table 2:** The immunohistochemical characteristics of the most common pediatric renal tumors with an epithelial pattern.

Epithelial Pattern	Immunohistochemistry								Ref
	WT1 (n)	AMACR	Pankeratin	CK7	TFE3	TFEB	Melanocytic markers	BRAF V600E	
**WT**	++	−−	+/−	−−	−−	−−	−−	−−	[16,17,18,19,20,21,22,23,24,25]
**NR**	+	NA	+	−/+ *	NA	NA	NA	−	[17,18]
**MA**	++	−	+/−	−/+ *	NA	NA	NA	+	[17,18,20,21,22,25,26]
**Xp11.2 tRCC**	−−	++	−/+ *	−	++	−−	−/+	−−	[14,24,26,27,28,29,30]
**t(6;11) RCC**	−−	+	−	−	−−	+	+	−−	[14,24,26,27,28,29,30]
**PRCC**	−−	++	++	+	−−	−−	−−	−−	[17,20,21,26,27,28]

WT = Wilms tumor; NR = nephrogenic rest; MA = metanephric adenoma; RCC = renal cell carcinoma; tRCC = translocation associated RCC; PRCC = papillary RCC; n = nuclear; NA = not available; * = if positive, often only focally; scoring: ++ >95% positive cases; + 76–95% positive cases; +/− 51–75% positive cases; −/+ 26–50% positive cases; − 5–25% positive cases; −− <5% positive cases.

**Table 3:** The immunohistochemical characteristics of the most common pediatric renal tumors with a mesenchymal pattern.

Mesenchymal Pattern	Immunohistochemistry							Notes	Ref
	WT1 (n)	NGFR	BCOR	Cyclin D1	INI1	CD34	BCL-2		
**WT**	+/−	−	−/+ *	−	++	−/+	+	Rhabdomyoblastic cells desmin +	[16,23,31,32,33,34,35,36,37]
**CCSK**	−−	++	+	++	++	−−	−/+		[16,31,32,33,34,35,36,37,38,39]
**Cellular/Mixed CMN**	−−	− *	− *	+/++	++	−−	−−		[16,19,31,32,33,34,35,36,37,40,41]
**Classic CMN**	−−	−−	− *	+/++	++	−−	−−		[16,19,31,34,35,36,37,40,41]
**MST**	+/−	−	− *	NA	++	++	NA	BRAFV600E +/−	[33,34,42,43]

WT = Wilms tumor; CCSK = clear cell sarcoma of the kidney; CMN = congenital mesoblastic nephroma; MST = metanephric stromal tumor; n = nuclear; c = cytoplasmic; var = variable; m = membranous staining; NA = not available; * = if positive, often only focally. Scoring: ++ >95% positive cases; + 76–95% positive cases; +/− 51–75% positive cases; −/+ 26–50% positive cases; − 5–25% positive cases; −− <5% positive cases.

**Table 4:** The immunohistochemical characteristics of the most common pediatric tumors with an undifferentiated pattern.

Undifferentiated Pattern	Immunohistochemistry										Notes	Ref
	WT1 (n)	INI1	NGFR	Keratin	CD99	NE	Desmin	PAX8	NB84	Cyclin D1		
**WT**	+ ^a^	++	-	+/−	−	−−	−	++	−−	−		[16,17,23,31,34,36,40,44,45,46,47,48,49,50,51]
**MRTK**	− (c +/−)	−−	− *	+/− (d) *	+/−	NA	+/− ^c^	−−	NA	++		[15,16,31,34,36,40,46,47,48,52]
**NB**	−	++	+	−	−−	++	−−	−−	++	+	PHOXB2 + in >90%	[16,31,40,47,48,50,51,53]
**DSRCT**	+ ^b^	++	−−	+ (d)	−	−	++ (d)	−−	−	−/+		[16,34,36,40,44,47,48,50,51]
**EWS**	−−	++	−	− **	++ (m)	−	−−	−−	−/+	++	Fli1+ in >85%	[16,36,45,47,48,49,50,51]

WT = Wilms Tumor; MRTK = malignant rhabdoid tumor of the kidney; NB = neuroblastoma; DSRCT = desmoplastic small round cell tumor; EWS = Ewing sarcoma; n = nuclear; NE markers = neuroendocrine markers; m = membranous staining; d = dotlike paranuclear staining; c = cytoplasmic; a = both N-terminal and C-terminal clone; b = C-terminal clone only; if positive, often only focally; * rarely diffuse positive staining; scoring: ++ >95% positive cases; + 76–95% positive cases; +/− 51–75% positive cases; −/+ 26–50% positive cases; − 5–25% positive cases; −− <5% positive cases.

**Table 5:** The most common genetic aberrations useful in the differential diagnoses of pediatric renal tumors.

Tumor Type	Subtype	Genetic Aberrations Useful in Diagnostics	Techniques
**WT**		~35% *WT1, CTNNB1, WTX*	NGS
		~10% *SIX1/2*	NGS
		~15% microRNA processing genes (*DROSHA, DGCR8, DICER1*)	NGS
		~ 2.5% *TRIM28* mutations (up to 90% in pure epithelial WT)	NGS
		~5% *TP53* mutations (Anaplastic WT)	IHC, NGS
		Less common mutations: *FXBW7, MYCN, BCOR, MLLT1*	NGS
**CCSK**		Somatic *BCOR*-ITD (85–100%)	IHC, FISH
		t(10;17) (~10%)	FISH
		Rare: *BCOR-CCNB3* translocation	FISH, NGS
**MRTK**		~95% biallelic inactivation *INI1* (*SMARCB1*) ~5% *SMARCA4* mutations	IHC, NGS, NGS
**CMN**	**Classic**	~57% *EGFR*-ITD	NGS
	**Cellular**	70–80% t(12;15)(p13;q25)	FISH
		12% *EGFR*-ITD	NGS
		Few cases reported with *BRAF*-ID rearrangements	NGS
	**Mixed**	t(12;15)(p13;q25)	FISH, NGS
		82% *EGFR*-ITD	NGS
**RCC**	**t(6;11) tRCC**	Translocations involving *TFEB*	IHC (TFEB), FISH
	**Xp11.2 tRCC**	Translocations involving *TFE3*	IHC (TFE3), FISH
	**PRCC**	Type 1: 81% *MET* alterations	NGS
		Type 2: 25% *CDKN2A* alterations	NGS
	**ALK-RCC**	Translocations involving *ALK*	FISH
	**HLRCC-RCC**	*FH* mutations	IHC, NGS
	**SDH-related RCC**	*SDHB* mutations	IHC, NGS
**Metanephric tumors**	**MA**	~90% *BRAFV600E* mutation	IHC, NGS
	**MST**	~65% *BRAFV600E* mutation	IHC, NGS
	**MAF**	~50% *BRAFV600E* mutation	IHC, NGS
**Neuroblastoma**		Mainly in high risk: *MYCN* amplification (18–38%)	FISH, NGS
		Mutations in a variety of genes (e.g., *MYCN)*	NGS
**EWS**		Translocations involving *EWS*	FISH
**DSRCT**		>95% t(11;22)(p13;q11.2 or q12)	FISH

WT = Wilms tumor; CCSK = clear cell sarcoma of the kidney; MRTK = malignant rhabdoid tumor of the kidney; CMN = congenital mesoblastic nephroma; RCC = renal cell carcinoma; tRCC = translocation associated renal cell carcinoma; ALK-RCC = anaplastic lymphoma kinase-rearranged RCC; HLRCC-RCC = hereditary leiomyomatosis renal cell carcinoma - RCC; SDH-related -RCC = succinate dehydrogenase related renal cell carcinoma; MA = metanephric adenoma; MST = metanephric stromal tumor; MAF = metanephric adenofibroma; EWS = Ewing sarcoma; DSRCT = desmoplastic small blue round cell tumor; IHC = immunohistochemistry; FISH = fluorescence in situ hybridization; NGS = next generation sequencing; ITD = internal tandem duplication; ID = internal deletion.

**Table 6:** Recommended immunohistochemical panels in the differential diagnosis of pediatric renal tumors.

Panels	Immunohistochemical Stains Recommended in Panel		Additional Stains Which Can be Useful	
**Epithelial pattern**	WT1	CK7	PAX8	INI-1
	Pan-cytokeratin	TFE3 *	BCOR *	CD10
	Melanocytic markers	TFEB *	Cathepsin K	Vimentin
	AMACR	BRAFV600E *	ALK	CyclinD1
			2SC/FH	
**Mesenchymal pattern**	WT1	BCL-2	INI1	pan-Trk *
	CD34	NGFR *	CD99	TLE1 *
	Cyclin D1	BCOR *	BRAFV600E *	
**Undifferentiated pattern**	WT1	Neuroendocrine markers	CD56	BCOR *
	INI1	Cyclin D1	CD99	PHOX2B *
	Keratins	NB84 *	CD45	
	Desmin	NGFR *		

In bold the most important immunohistochemical stains per pattern which will distinguish the most important tumors in the differential diagnosis of each pattern; the additional markers can be used as further support for the differential diagnosis as described in the text and Table 2, Table 3 and Table 4; * if available.

**Table 7:** Translocations present in the most common pediatric renal tumors which can be demonstrated by fluorescent in situ hybridization (FISH).

Tumor Type	Translocation	Fusion	FISH Probe
**Xp11.2 tRCC**	t(X;17)(p11.2;q25)	*TFE3-ASPL*	*TFE3* break-apart
	t(X;1)(p11.2;q21)	*TFE3-PRCC*	
	t(X;1)(p11.2;p34)	*TFE3-PSF*	
	(X;X)(p11.2;q12)	*TFE3-NonO*	
	t(X;17)(p11.2;q23)	*TFE3-CLRC*	
**t(6;11) tRCC**	t(6;11)(p21;q12)	*TFEB-MALAT1*	*TFEB* break-apart
**CCSK**	t(10;17)(q22;p13)	*YWHAE-NUTM2B/E*	*YWHAE* or *NUTM2* break-apart
	inv(X)(p11.4;p11.22)	*BCOR-CCNB3*	*BCOR-CCNB3* fusion
**CMN**	t(12;15) (p13;q25)	*ETV6-NTRK3*	*ETV6* or *NTRK3* break-apart; *ETV6-NTRK3* fusion
**DSRCT**	t(11;22)(q13;q12)	*WT1-EWS*	*EWS* break-apart
**EWS-rearranged sarcomas**	t(11;22)(q13;q12)	*WT1-EWS*	*EWS* break-apart
	t(11;22)((q24;q12)	*FLI-1-EWS*	
	t(21;22)(q12q12)	*ERG-EWS*	
	t(7;22)(p22;q12)	*ETV1-EWS*	
	t(17;22)(q12;q12)	*E1AF-EWS*	
	t(2;22)(q33;q12)	*FEV-EWS*	
**EWS-negative sarcomas**	t(4;19) or t(10;19	*CIC-DUX4*	*CIC* or *DUX4* break-apart
	inv(X)(p11.4;p11.22)	*BCOR-CCNB3*	*BCOR-CCNB3* fusion
**ALK-RCC**	t(2;10)(p23;q22)	*VCL-ALK*	*ALK* break-apart
	t(1;2)(q25;p23)	*TPM3-ALK*	
	t(1;2)(p32;p23)	*HOOK-ALK*	

FISH = fluorescence in situ hybridization; tRCC = translocation associated renal cell carcinoma; CCSK = clear cell sarcoma of the kidney; CMN = congenital mesoblastic nephroma; DSRCT = desmoplastic small blue round cell; EWS = Ewing Sarcoma; ALK-RCC: anaplastic lymphoma kinase-rearranged RCC.

## Supplementary Materials

The following are available online at https://www.mdpi.com/2072-6694/12/3/729/s1, Table S1: Syndromes associated with the most common pediatric renal tumors; Table S2: Histopathological, immunohistochemical and genetic aberrations useful in the differential diagnosis of the most common pediatric renal tumors.
