# Supplementary material for: Renal Tumors of Childhood—A Histopathologic Pattern-Based Diagnostic Approach
Source: Cancers (Basel). 2020 Mar 19;12(3):729. doi: 10.3390/cancers12030729 (PMC7140051; doi:10.3390/cancers12030729)
Supplement: Supplementary file 1 [file cancers-12-00729-s001.pdf]

Supplementary Materials:

Table S1. Syndromes associated with pediatric renal tumours.

Tumor	Tumor subtype	Associations with other disease/syndromes
**WT**	All subtypes	Beckwith Wiedemann-syndrome (loss of imprinting at 11p15)<br>Bohring-Opitz syndrome (*ASXL1* mutations)<br>Denys-Drash-syndrome (*WT1* missense mutations)<br>Familial nephroblastoma (loci FWTS1 and FWTS2 (likely *TRIM28*))<br>Fanconi anemia (*BRCA1* and *PALB2* mutations)<br>Frasier syndrome (*WT1* splicing mutations)<br>GLOW (Global developmental delay, Lung cysts, Overgrowth, WT)(*DICER1* mosaic)<br>Isolated hemihyperplasia (11p15 imprinting abnormalities)<br>Li–Fraumeni syndrome (*TP53* mutations)<br>Mulibrey (muscle-liver-brain-eye) nanism (*TRIM37*)<br>Perlman syndrome (*DIS3L2* mutations)<br>Simpson–Golabi–Behmel (*GPC3* mutations)<br>WAGR-syndrome (Wilms tumor, Aniridia, Genitourinary malformations and mental Retardation; microdeletion *WT1* and *PAX6*)
**CCSK**		None
**MRTK**		~33% Familial Rhabdoid Predisposition syndrome (*SMARCB1*(*INI1*) mutation, rarely *SMARCA4* mutation)
**CMN**	**Classic (~30%)**	None
	**Cellular**	None
	**Mixed**	None
**RCC**	**Xp11.2 tRCC (TFE3-rearranged tRCC)**	None
	**t(6;11) tRCC (TFEB-rearranged tRCC)**	None
	**PRCC**	Hereditary papillary renal carcinoma (AD) due to germline-activating mutations *MET* gene
	**ALK-tRCC**	Sickle cell trait in children with *VCL-ALK* (100%)
	**HLRCC-RCC**	Almost all RCCs with FH- by immunohistochemistry have *FH* germline mutation associated with HLRCC (AD)<br>Penetrance for RCC 20-30%
	**SDH-RCC**	Germline *SDH* mutations associated with Hereditary Paraganglioma-Pheochromocytoma Syndrome<br>Pediatric cases: *SDHB* mutations, in all tested cases germline mutations present

**Metanephric tumors**	**MA**	Polycythemia
	**MST**	None
	**MAF**	None
**Neuroblastoma**	**Poorly differentiated/ undifferentiated**	1-2% familial (AD): mutations *ALK, PHOX2B, KIF1Bβ, RAS* pathway mutations (Costello syndrome (*HRAS*), Noonan syndrome (mainly *SOS1*), Neurofibromatosis type 1 (*NF1*))<br>Li–Fraumeni syndrome (*TP53* mutations)<br>Hereditary pheochromocytoma/paraganglioma syndrome (*SDH* mutations)
**EWS**		Mutations in DNA repair pathway genes
**DSRCT**		None

WT = Wilms tumor; CCSK = clear cell sarcoma of the kidney; MRTK = malignant rhabdoid tumor of the kidney; CMN = congenital mesoblastic nephroma; RCC = renal cell carcinoma; tRCC = translocation associated renal cell carcinoma; PRCC = papillary RCC; ALK-RCC = anaplastic lymphoma kinase-translocation RCC; HLRCC = hereditary leiomyomatosis renal cell carcinoma; SDHB deficiency-associated RCC = succinate dehydrogenase (B)-associated renal cell carcinoma; MA = metanephric adenoma; MST = metanephric stromal tumor; MAF = metanephric adenofibroma; EWS = Ewing sarcoma; DSRCT = desmoplastic small round cell tumor; AD = autosomal dominant

Table S2. Histopathologic features useful in differential diagnosis of pediatric renal tumors

Tumor	Tumor subtype	Histology
**WT**		3 components (highly variable proportions): blastema, stromal, and epithelial, ~5-10% show anaplasia (nuclear hyperchromasia, nuclear enlargement, and atypical mitoses), focal or diffuse, 40% associated with nephrogenic rests (precursor)<br>Europe (SIOP)-histologic classification for treatment stratification after pre-operative chemotherapy is based on the percentage of chemotherapy-induced changes, percentage of viable components, and the presence of diffuse anaplasia.<br>USA (COG)-histologic classification based solely on the presence of anaplasia, after primary nephrectomy.
**CCSK**		Classic pattern (~90% at least focal):<br>Nests/cords of ovoid, spindled or epithelioid cells containing bland nuclei<br>Extensive fibrovascular ("chicken wire") network<br>Less frequent patterns: myxoid (50%), sclerosing (35%), cellular (26%), epithelioid (13%), palisading (11%), spindle-cell (7%), storiform (4%), and anaplastic (3%)
**MRTK**		Non-encapsulated tumor, hemorrhage and necrosis present<br>Classic pattern: infiltrating sheets of non-cohesive cells, large eccentric nucleus, abundant eosinophilic cytoplasm, high mitotic activity
**CMN**	**Classic (~30%)**	Bland spindle cells, collagenous stroma, interlacing fascicular pattern
	**Cellular**	High cellularity, ovoid cells with less cytoplasm, more solid sheets, higher proliferation rate
	**Mixed**	Areas of both classic and cellular type
**RCC**	**Xp11.2 tRCC (TFE3-rearranged tRCC)**	Large epithelioid cells, prominent nucleoli, clear to eosinophilic cytoplasm<br>*ASPL-TFE3 fusion*: papillary or nested pattern;<br>*PRCC-TFE3 fusion*: smaller nests, some papillae, less cytoplasm, less conspicuous nucleoli; psammoma bodies often present; mitoses rare
	**t(6;11) tRCC (TFEB-rearranged tRCC)**	Biphasic pattern with nests of large epithelioid cells with clear to eosinophilic cytoplasm mixed with smaller cells surrounding basement membrane material
	**PRCC**	Type 1: small cuboidal cells, scant cytoplasm, arranged as single layers on papillae, tubules and glomeruloid structures, often foamy macrophages and psammoma bodies<br>Type 2: papillae with pseudostratified cells, abundant eosinophilic cytoplasm, atypical nuclei with prominent nucleoli
	**ALK-tRCC**	Sheets of polygonal to spindle-shaped cells with large vesicular nuclei and abundant eosinophilic cytoplasm<br>Characteristic intracytoplasmic lumina<br>Presence of a lymphoplasmacytic infiltrate
	**HLRCC-RCC (Fumarate Hydratase deficient RCC)**	Most commonly mixed patterns, including papillary, tubulo-papillary, tubular, and solid<br>Hallmark: viral inclusion-like eosinophilic nucleolus surrounded by a clear halo
	**SDH-tRCC**	Solid architecture, sometimes nested or cystic<br>Eosinophilic cytoplasm; inconspicuous nucleoli, intracytoplasmic vacuoles<br>Hallmark: flocculent inclusions (can be focal)

**Metanephric tumors**	**MA**	Sharply demarcated, unencapsulated tumor<br>Uniform closely packed tubules and papilla, glomeruloid bodies<br>Small cells with scant cytoplasm, overlapping bland nuclei with fine chromatin<br>No atypia; rare mitoses<br>Psammoma bodies often numerous
	**MST**	Non-encapsulated tumor; entrapment surrounding native kidney with "onion skinning" around tubules and angiodysplasia<br>Alternating areas of hypo- and hypercellular areas;<br>Bland spindled cells, indistinct cytoplasm, rare mitosis<br>May show heterologous differentiation
	**MAF**	Composite of both MA and MST components in highly variable proportions
**Neuro-blastoma**	**Poorly differentiated/ undifferentiated**	Nuclei of undifferentiated cells with characteristic 'salt and pepper' chromatin
**EWS**		Small round to oval cells with scant cytoplasm<br>Condensation around blood vessels<br>Neuroepithelial features<br>Often originates from retroperitoneum with infiltration of kidney
**DSRCT**		Solid nests of small blue round to oval cells, mostly embedded in desmoplastic stroma<br>Necrosis and cystic degeneration<br>Pseudoglandular and pseudorosette formation<br>Rare rhabdoid cells

WT = Wilms tumor; CCSK = clear cell sarcoma of the kidney; MRTK = malignant rhabdoid tumor of the kidney; CMN = congenital mesoblastic nephroma; RCC = renal cell carcinoma; tRCC = translocation associated renal cell carcinoma; PRCC = papillary RCC; ALK-RCC = anaplastic lymphoma kinase-translocation RCC; HLRCC = hereditary leiomyomatosis renal cell carcinoma; SDHB deficiency-associated tRCC = succinate dehydrogenase B-associated renal cell carcinoma;MA = metanephric adenoma; MST = metanephric stromal tumor; MAF = metanephric adenofibroma; EWS = Ewing sarcoma; DSRCT = desmoplastic small round cell tumor
